# Five Hours of Resuscitation With 150 Electrical Shocks and Complete Recovery

**DOI:** 10.7759/cureus.14255

**Published:** 2021-04-02

**Authors:** Vsevolod Tabachnikov, Keren Zissman, Hussein Sliman, Moshe Y Flugelman

**Affiliations:** 1 Cardiology, Carmel Medical Center, Haifa, ISR

**Keywords:** cardiac arrest, acute coronary syndromes, resuscitation

## Abstract

Background: Myocardial ischemia may lead to lethal arrhythmias. Treatment of these arrhythmias without addressing the cause of ischemia may be futile. The length of resuscitation is an important parameter for determining when to stop resuscitation but with shockable rhythms and reversible cause of the cardiac arrest, the decision to terminate resuscitation is complex.

Case Summary: A patient with a three-month history of shortness of breath with effort developed pulseless ventricular tachycardia (VT) at the early stages of a stress test. In coronary angiography, a critical lesion in the right coronary artery (RCA) was observed and treated with two stents. During the procedure and for a total of five hours, the patient had more than 100 separate episodes of VT and ventricular fibrillation (VF) that were treated by 150 defibrillations, artificial ventilation, intra-aortic counter-pulsation balloon insertion, and multiple drugs. One hour after the initial stenting procedure, thrombosis of the RCA was demonstrated and treated successfully with angioplasty. Use of procainamide resolved the arrhythmias and the patient recovered completely without neurological deficit, ejection fraction of 45%, and is asymptomatic at one year following the event.

Discussion: Our case shows that with a revisable cause of cardiac arrest, resuscitation should be directed at maintaining perfusion of essential organs and treating the reversible cause. Without re-opening the RCA, we could not have saved the patient's life. The use of an extracorporeal membrane oxygenator, if available, should be considered in similar cases. Finally, the quality of cardiopulmonary resuscitation determines the neurological outcome regardless of the length of resuscitation, as was evident in our patient who recovered completely.

## Introduction

When myocardial ischemia leads to lethal arrhythmias treating these arrhythmias without treating the cause of ischemia may be futile. Prompt coronary reperfusion should be the major treatment objective and no delays are acceptable [1]. Patients presenting with recurrent lethal arrhythmias (arrhythmic storm) should be stabilized to be able to undergo angiography and revascularization [2,3]. Stabilization may delay the timing of revascularization and any treatment should be weighted in this respect.

Length of resuscitation is an important parameter for the decision to stop resuscitation, but with shockable rhythms and reversible cause of the cardiac arrest, the decision to terminate resuscitation is complex regardless of the length of resuscitation [4,5]. The case report we present demonstrates several management dilemmas and the importance of revascularization integrated with quality resuscitation to maintain essential organ perfusion. 

## Case presentation

**Figure 1:**
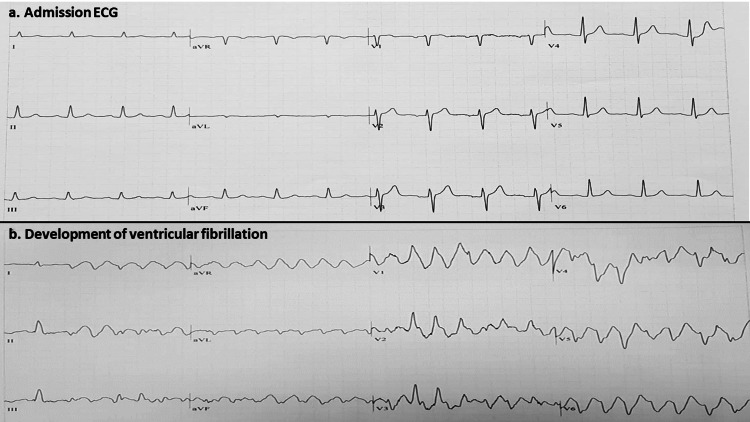
Admission electrocardiogram and an electrocardiogram of an episode of ventricular fibrillation a. Admission electrocardiogram: normal sinus rhythm, no signs of ischemia and no QT segment prolongation. b. An episode of polymorphic ventricular tachycardia - ventricular fibrillation.

A 66-year-old patient was admitted to our cardiac intensive care unit (CICU) after developing pulseless ventricular tachycardia (VT)-ventricular fibrillation (VF) during an exercise test. He was defibrillated promptly and arrived in the CICU with no complaints, hemodynamically stable, and no ischemic electrocardiogram (ECG) changes. Three months prior to the index hospitalization, he had started complaining of shortness of breath on effort. He had a history of paroxysmal atrial fibrillation and was treated with bisoprolol and flecainide after failed ablation for atrial fibrillation. His baseline ECG showed no signs of ischemia and his QT was normal (Figure 1a). Shortly after starting the exercise test, the patient complained of angina and immediately lost consciousness and his ECG showed polymorphic VT-VF (Figure 1b).

**Figure 2:**
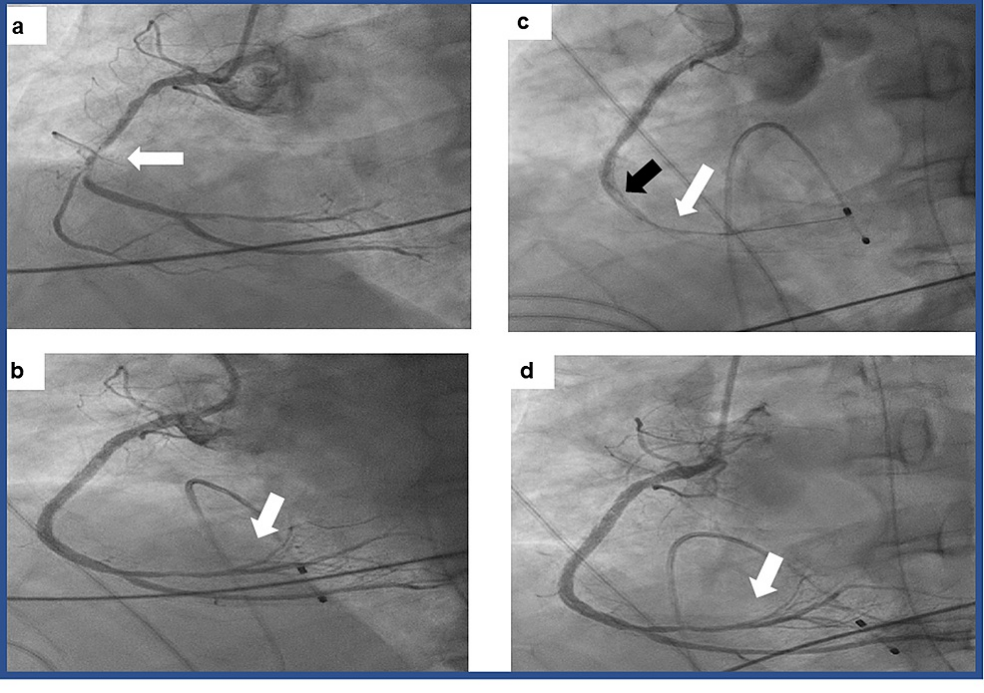
Angiographic findings a. Coronary angiography showing a critical long narrowing of the right coronary artery (white arrow) (time 0). b. Coronary angiography showing patent right coronary artery after stents implantation with filling of the distal coronary branches of the artery (white arrow) (time 0.5 hours). c. Coronary angiography showing the right coronary artery with in-stent thrombosis as indicated by the filling defect (black arrow) and no filling of distal artery (white arrow) (time 4 hours). d. Coronary angiography showing the right coronary artery with patent stents after balloon dilatation and good filling of the distal artery (white arrow) (time 4.5 hours).

In the CICU, he received a loading dose of aspirin and clopidogrel, and 6 hours after arrival, he underwent coronary angiography using radial access. A critical narrowing was observed in the proximal right coronary artery (RCA) with no angiographic evidence of thrombus (Figure 2a). Two drug-eluting stents were implanted with a good angiographic result (Figure 2b) and thrombolysis in myocardial infarction (TIMI) grade 3 flow. During coronary angiography and angioplasty, ST elevation was noted in inferior ECG leads and multiple events of pulseless VT-VF were observed and were treated with defibrillations. importantly, there were no signs of the "no-reflow" phenomenon and no pressure dampening during catheterization and the intervention in the RCA. At the end of the procedure, the patient was asymptomatic, hemodynamically stable, and with no arrhythmia and was transferred to the CICU.

Thirty minutes after arrival to the CICU, the patient developed recurring events of pulseless VT and events of VF that required cardiac massage, multiple defibrillations, adrenaline (total of 3 mg), amiodarone (300 mg loading dose), and lidocaine (100 mg loading dose). The patient was intubated, and ventilation was initiated but recurrent VT and VF leading to arrhythmia storm necessitated continuous cardiac massage and defibrillations. To control the VT-VF storm, an intra-aortic counter-pulsation balloon was inserted at the bedside. Electrolytes were normal and blood gases showed good oxygenation. The frequency of VT-VF was under some control enabling us to transfer the patient 1 hour after the initial coronary intervention back to the catheterization laboratory in which recurrent thrombosis of RCA was demonstrated (Figure 2c). While performing cardiac massage, balloon expansion of RCA was performed which restored TIMI grade 3 flow in the artery (Figure 2d). Despite restoring TIMI grade 3 flow in the RCA, VT and VF reoccurred and required defibrillations and cardiac massage. To control arrhythmia after the failure of the aforementioned measures and drugs, we treated the patient with two sequential doses of 100 mg procainamide each intravenously, and the arrhythmias ceased after injecting 200 mgs. Overall, the patient received more than 150 defibrillations and the total time of actual resuscitation was 5 hours starting from the first stenting procedure and the cessation of arrhythmia after the second angioplasty and treatment with procainamide. Twenty-four hours later, the patient was awake, extubated, and with no neurological deficits. The ejection fraction on echocardiography improved from 35% after the resuscitation to 45% one week later with inferior wall hypokinesia and normal right ventricular function. No arrhythmias were observed. The patient was discharged home with 7.5 mg bisoprolol, 2.5 mg ramipril, ticagrelor 90 mg twice a day, 100 mg aspirin, and atorvastatin 80 mg. He is asymptomatic 1 year later and works a full-time job. 

## Discussion

The patient we describe was treated for recurrent VT and VF in the context of acute coronary syndrome (ACS). The patient exhibited many features that are common in ACS, namely lethal arrhythmia induced by myocardial ischemia, recurrent thrombosis of the coronary arteries, and recovery after coronary occlusions got treated. The time from the first defibrillation in the catheterization laboratory till the time of cessation of arrhythmia after the second coronary intervention and injection of procainamide was 5 hours and included the two coronary interventions and 1 hour in the CICU between the two interventions. Complete recovery after 5 hours of resuscitation and 150 defibrillations is highly uncommon. Although intuitively we can correlate longer resuscitations with poor survival and neurological outcome, in a large study, it was shown that patients with longer resuscitations in the hospital have higher rates for return of spontaneous circulation and discharge [6]. Obviously, in this study, the patients were not resuscitated for 5 hours.

Two options were considered during resuscitation, the first was the use of mechanical cardiac massage and the second was the use of extracorporeal membrane oxygenation (ECMO) [7]. Simple mathematics shows that during the 5 hours of resuscitation, excluding short intermissions with sinus rhythm and time of transfer and preparations for catheterization, we treated with drugs and defibrillations lethal arrhythmias every 1.5-2 minutes. Since we assumed that coronary occlusion was the drive for the arrhythmias, we aimed at getting to the catheterization laboratory at the earliest possible time and therefore we decided not to pursue the option of ECMO that could delay coronary reperfusion in the catheterization laboratory. The complete neurological recovery and the near-normal ejection fraction attest to the quality of the cardiopulmonary resuscitation (CPR) [4,5]. The outcomes of mechanical cardiac massage were shown to be inferior to those of manual cardiac massage [8]. If quality manual cardiac massage can be performed, as was the case in our patient, it should be employed, but additional studies are needed to determine the efficiency of mechanical cardiac massage. The use of 12 leads electrocardiogram immediately after resuscitation is of limited value to diagnose total occlusion of coronary arteries [9] based on ST segment elevation. Due to the multiple VF events, 12 leads electrocardiogram was not available, but our understanding that re-occlusion of the RCA could occur during resuscitation or could cause the recurrent arrhythmic events drove us to perform the second coronary angiography. 

A major issue that was relevant to our patient is resuscitation termination. Apparently, there are no clear rules for stopping resuscitation. However, in patients with asystole and no reversible cause, the timing of termination is dictated by the failure of return of spontaneous circulation. In a patient with shockable rhythms and a reversible cause, as was the case with our patient, termination is usual after a prolonged period and failure to control the arrhythmia despite the use of several drugs and after intubation [4]. The sequence of events in our patient highlights the need for use of multiple measures and drugs combined with treatment of reversible cause regardless of the length of resuscitation. 

## Conclusions

With a reversible-cause dysrhythmia, quality of resuscitation and rapid transfer to the catheterization laboratory when indicated determines the neurological outcome regardless of the length of resuscitation, as was evident in our patient, who recovered completely.
